# Iron Metabolism in Field Hockey Players During an Annual Training Cycle

**DOI:** 10.1515/hukin-2015-0066

**Published:** 2015-10-14

**Authors:** Tomasz Podgórski, Jakub Kryściak, Jan Konarski, Katarzyna Domaszewska, Krzysztof Durkalec-Michalski, Ryszard Strzelczyk, Maciej Pawlak

**Affiliations:** 1 Department of Biochemistry, University School of Physical Education in Poznan, Poland.; 2 Department of Physiology, University School of Physical Education in Poznan, Poland.; 3 Department of the Theory of Sport, University School of Physical Education in Poznan, Poland.; 4 Department of Hygiene and Human Nutrition, Dietetic Division, Poznań University of Life Sciences, Poznan, Poland.

**Keywords:** TIBC, UIBC, aerobic fitness, haematology

## Abstract

Post-physical training changes in iron metabolism in the human body often occur. To fully describe these processes, fifteen male Polish National Team field hockey players (age 27.7 ± 5.2 years, body mass 72.8 ± 7.6 kg and body height 177.1 ± 5.7 cm) were examined in three phases of an annual training cycle: preparatory (T1), competitive (T2) and transition (T3). To assess aerobic fitness, maximal oxygen uptake (VO_2_max) was evaluated. Based on the iron concentration, the changes in total iron binding capacity (TIBC), unsaturated iron binding capacity (UIBC) and other selected haematological indicators (haemoglobin, erythrocytes, mean corpuscular haemoglobin - MCH) in iron metabolism were estimated. The average values of maximum oxygen uptake increased from 54.97 ± 3.62 ml·kg^−1^·min^−1^ in T1 to 59.93 ± 3.55 ml·kg^−1^·min^−1^ in T2 (p<0.05) and then decreased to 56.21 ± 4.56 ml·kg^−1^·min^−1^ in T3 (p<0.05). No statistically significant changes in the erythrocyte count were noted. The MCH and haemoglobin concentration decreased between T1 and T2. The maximal exercise test caused a significant (p<0.05) increase in the plasma iron concentration during the competition and transition phases. Progressive but non-significant increases in resting iron concentration, TIBC and UIBC in the analysed annual training cycle were noted. To show global changes in iron metabolism in the human body, it is necessary to determine additional variables, i.e. UIBC, TIBC, haemoglobin, MCH or the erythrocyte count. The direction of changes in iron metabolism depends on both the duration and intensity of the physical activity and the fitness level of the subjects. Dietary intake of iron increases the level of this trace element and prevents anaemia associated with training overloads.

## Introduction

Every physical activity causes changes in the metabolic processes at the cellular level. This process is confirmed by changes in the concentration of a number of biochemical variables and the level of haematological components, particularly observed following high intensity effort (Green et al., 1984).

One of the important variables that determine aerobic fitness in athletes is the concentration and availability of iron in the body. This particularly affects endurance sports, which also include field hockey. Iron is a cellular component of oxidative phosphorylation (cytochromes) as well as a fundamental element of endurance and strength developed in the muscles of athletes due to the activity of antioxidant enzymes (Schneider et al., 2005). This biochemical microelement, as a component of haemoglobin, mediates and provides a marker of blood oxygen capacity; the concentration of haemoglobin in the blood has an effect on the results achieved by athletes (Wehrlin et al., 2006). Muscle myoglobin, apart from ensuring oxygen during intensive physical exercise, exhibits antioxidant properties and acts as an enzyme, similarly to other enzymes with an iron atom in their structure, i.e. peroxidase and catalase; these protect skeletal muscles (Brunori, 2001) and the myocardium (Flögel et al., 2004) against the damaging effects of free radicals (NO).

Numerous studies on iron metabolism including the metabolism levels, the concentration of haemoglobin, or the erythrocyte count, do not describe the conditions of competitive sport (Worwood, 1997). Iron metabolic diagnostic potential can only be increased significantly following the application of additional variables that are typical of the turnover of this compound in the body; these include TIBC (total iron binding capacity), UIBC (unsaturated iron binding capacity), transferrin and ferritin. The great importance that the distribution of iron plays in the healthy functioning of body systems suggests the need for systematic monitoring of this compound over the entire training cycle. Single measurements of the previously mentioned biochemical and haematological parameters are of very limited diagnostic value when considering iron metabolism in the body of athletes.

Total iron binding capacity describes the amount of iron required for transferrin saturation in the serum. For this reason, medical diagnostics use TIBC and the concentration of transferrin interchangeably (Yamanishi et al., 2002). Low saturations of TIBC can indicate iron overload; however, high levels demonstrate iron deficiency in the body (Peter and Wang, 1981). This is usually related to insufficient iron accumulation from nutrition together with greater demand during physical exercise (Beard and Tobin, 2000). Low concentrations of iron in the body can also lead to heme synthesis imbalance, which may effect a poor oxygen transport to contracting muscles and reduced effectiveness of the weight-training exercise (Deruisseau et al., 2004).

A biochemical indicator with similar diagnostic potential as TIBC includes the unsaturated iron binding capacity. This parameter describes the amount of iron that can saturate free transferrin circulating in the blood. The concentration of UIBC, which in a group of healthy Caucasian male accounts for approximately 70% of the total binding capacity of iron (McLaren et al., 2001), increases when there is an iron deficiency or decrease (haemorrhage); however, UIBC decreases during haemolytic anaemia, chronic infection and liver disease.

It is generally accepted that ensuring appropriate levels of iron concentration, TIBC, haemoglobin and the optimum erythrocyte count required by the body of an athlete should be taken into consideration as an important issue of planned training. The aim of this study was to assess the changes in the biochemical indicators related to iron metabolism in the blood of field hockey players in an annual training cycle.

## Material and Methods

### Participants

Fifteen male Polish national field hockey players (age 27.7 ± 5.2 years), excluding goalkeepers, were involved in this study. The somatic characteristics and training experience are presented in Table 1. No significant differences in body mass and the BMI in the annual training cycle were found. The study commenced following approval from the Bioethics Committee of the University of Medicine in Poznań (Poland). It included a full training cycle planned for field hockey players from the preparatory phase (T1), through the competitive phase (T2) until the transition phase (T3). The investigated athletes were on a balanced diet that was not enriched with iron supplements.

### Training loads

The training sessions were divided into three phases: (i) Preparatory (T1, 8 weeks); (ii) Competitive (T2, 6 weeks); and (iii) Transition (T3, 4 weeks). The preparatory phase, including general and specific exercises, involved training loads that were appropriate to the current fitness level of competitors. In this phase, training consisted mainly of aerobic and resistance exercises, which later provided the foundation for high intensity training related to field hockey. Endurance training loads were estimated on the level of the anaerobic threshold. In the preparatory phase, the volume and intensity of training increased gradually from 10 to 14 hrs divided into 6 to 8 training sessions per week, including 1 to 2 scrimmage matches weekly in the last 3 weeks of this phase.

In the competitive phase, the training loads (volume and intensity) were changed according to the game schedule. This training phase included mainly aerobic power and speed training (6 hrs divided into 4 training sessions per week). Moreover, highly specified training related to field hockey in the competitive phase was realized (4–5 hrs divided into 3 training sessions per week). Two competitive matches weekly (usually Saturday and Sunday) were played.

Training in the transition phase was directed towards regeneration and preparation of athletes for the upcoming training cycle; 5–6 hrs of training divided into 4–5 training sessions per week consisted mainly of aerobic and light strength exercises.

### Physiological Assessment

The exercise tests were conducted between 8:00 a.m. and noon, in an air-conditioned laboratory, 2 hours after consuming a light breakfast. Athletes underwent a graded exercise test to volitional exhaustion, which included running on a treadmill (Woodway, USA) with a starting speed of 8 km/h that was increased every 3 min by 2 km·h^−1^ until volitional exhaustion. Expired gases, minute ventilations (Ve) and a heart rate (HR) during the exercise protocol were monitored continuously by an automated system (Oxycon Mobile; Viasys Healthcare, Hoechberg, Germany). Oxygen intake (VO_2_) was measured breath-by-breath and was averaged at 15-s time periods. Before each trial, the system was calibrated according to the manufacturer’s instructions. Maximal oxygen uptake (VO_2_max) was defined as the highest 15 s averaged VO_2_ obtained during the last exercise load of the test. To determine the optimal endurance training loads, the anaerobic threshold (ventilatory threshold, V-slope method) was estimated.

### Blood collection and analysis

Before testing, 10 ml of blood was obtained from the ulna vein of each competitor and capillary blood was obtained from the fingertip (100 μl). Three minutes after the exercise protocol, capillary blood (100 μl) was again obtained. Moreover, 10 minutes after the cessation of testing, 10 ml of venous blood was collected to determine the serum iron concentration.

Lactate concentration was determined in capillary blood using the spectrophotometric enzymatic method (Maughan, 1982). The concentration of haemoglobin, the erythrocyte count and mean corpuscular haemoglobin (MCH) were measured in venous blood using a haematological analyser (Coulter MD2, Sysmed Lab, USA). The spectrophotometric colorimetric method described by Stookey (1970) and later modified by Persijn (1971) was used to determine the total iron concentration and UIBC. TIBC, recorded as μg·dL^−1^ of blood serum, was calculated as the sum of total iron concentration and UIBC.

### Statistical analysis

In addition to the descriptive statistics, differences between the pre- and post-exercise variables among the three observed phases (preparatory, competition and transition) were compared with ANOVA and the signed rank Wilcoxon test for pairwise comparisons. The Statistica software package (version 9.1, StatSoft, Inc., 2010) for MS Windows was used. The results were presented as the means and standard deviations (SD). The level of significance was set at p<0.05.

## Results

The average values of maximum oxygen uptake increased from 54.97 ± 3.62 ml·kg^−1^·min^−1^ in T1 to 59.93 ± 3.55 ml·kg^−1^·min^−1^ in T2, and then decreased to 56.21 ± 4.56 ml·kg^−1^·min^−1^ in T3. Mean values of VO_2_max registered in T2 were significantly higher (p<0.05) than in other training phases.

### Lactate concentration (LA)

The mean values of the pre-exercise LA concentrations ranged from 1.0 ± 0.2 mmol·L^−1^ in T1 to 1.5 ± 0.4 mmol·L^−1^ in T2 (T3; 1.3 ± 0.3 mmol·L^−1^). The exercise protocol caused significant (p<0.05) post-exercise increases in the concentration of this metabolite (8.4 ± 2.1 mmol·L^−1^, 7.8 ± 1.1 mmol·L^−1^, 7.6 ± 2.0 mmol·L^−1^; in T1, T2, T3, respectively). No statistically significant differences between the phases were observed. Almost identical post-exercise LA values indicate that the athletes performed with a similar level of volitional exhaustion.

### Values of haematological indicators

In Table 2, the results of the haematological indicators of iron metabolism are presented. The training loads between the preparatory and competitive phases led to a decrease in the values of haemoglobin and MCH.

### Iron concentration

**Figure 1:**
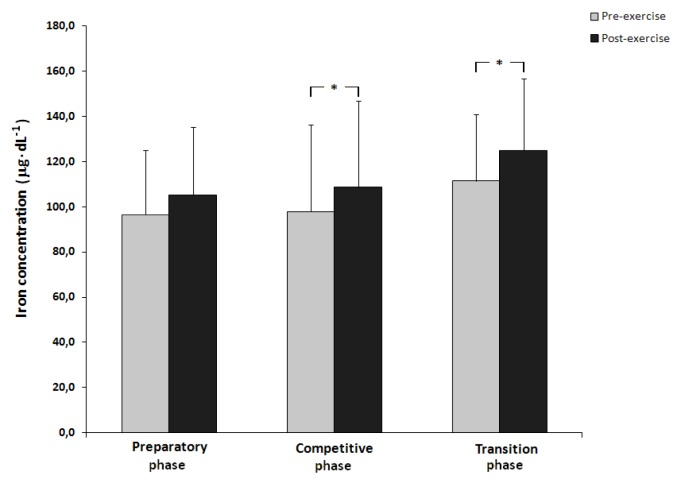
Pre- and post-exercise serum concentration of iron in field hockey players in three phases of an annual training cycle (* - p<0.05).

Resting iron levels detected in the blood serum increased with subsequent testing phases (T1, 96.40±28.62 μg·dL^−1^; T2, 97.80±38.41μg·dL^−1^; T3, 111.40±29.36 μg·dL^−1^). A significant increase in the concentration of iron was noted following physical tests in the competitive and transition phases (p<0.05) (Figure 1).

### Unsaturated and Total Iron Binding Capacity (UIBC & TIBC)

**Figure 2:**
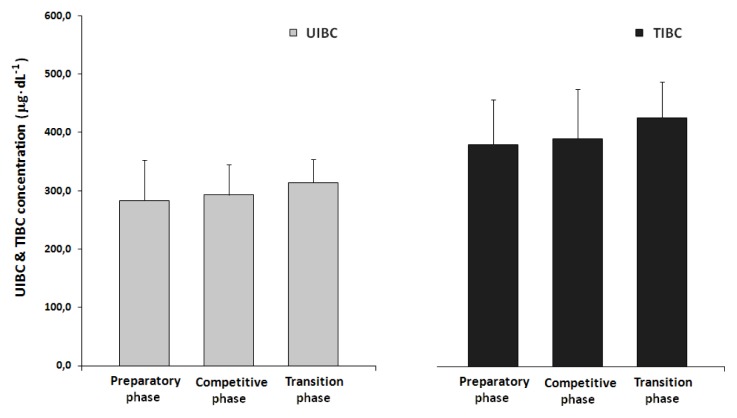
The pre- and post-exercise unsaturated total iron binding capacity (UIBC) and total iron binding capacity (TIBC) in the serum of field hockey players during three phases of an annual training cycle.

During the monitored training cycle, the resting concentrations of UIBC (T1, 283.07 ± 68.94μg·dL^−1^; T2, 292.80 ± 51.90μg·dL^−1^; T3, 314.00 ± 39.80μg·dL^−1^) and TIBC (T1, 379.47 ± 77.65μg·dL^−1^; T2, 390.60 ± 85.17μg·dL^−1^; T3, 425.40 ± 62.64μg·dL^−1^) increased. No statistically significant differences were observed (Figure 2).

## Discussion

In the available literature, there are very few publications on the metabolism of iron in field hockey athletes (Podgórski and Pawlak, 2011). Most of the topics covered in these articles involve women (Diehl et al., 1986) or consist of only one testing phase (Douglas, 1989). The authors of this study are the first to describe the metabolism of iron in field hockey athletes throughout an annual training cycle, using indicators of prevalent concentrations and the possible requirements for iron among men participating in professional field hockey.

Anthropometric data from tested field hockey players were very similar to those studied in other national teams i.e. the Republic of South Africa (Scott, 1991) and Switzerland (Telford et al., 1988). However, Polish field hockey players achieved higher values than team members from China and Malaysia (Demuth et al., 2007).

The results recorded during the maximal exercise tests on the treadmill, i.e. values of maximum oxygen uptake (approximately 55–60 ml·kg^−1^·min^−1^), were characteristic for elite field hockey players and other team sports competitors (Aziz et al., 2000). Changes in VO_2_max in the annual training cycle were typical for this type of sport discipline (Manna et al., 2009).

Long-term training between T1 and T2 caused reduced levels of resting haemoglobin and MCH (p<0.05), but did not influence the erythrocyte count. Similar results were described by Wilkinson et al. (2002); they indicated that intensive physical activity during a training cycle decreases the concentration of haemoglobin and other haematological variables. Magazanik et al. (1988) demonstrated that 7 week endurance training caused symptoms of sports anaemia. On the other hand, Barati (2009) showed no statistically significant changes in haematological variables in middle distance runners who underwent a 13 week training programme.

Very few publications describe the influence and long-term effects of field hockey training specifically on iron concentration. One study showed that training loads led to depletion of iron in the body of female competitors (Diehl et al., 1986). In our study, the highest values of resting iron concentration in the transition phase did not differ statistically from those recorded in T1 and T2. Comparable and mainly normal values of serum iron concentration during the entire training cycle showed no iron deficiency in tested athletes. No training effects on iron status in different groups of athletes (cross-country skiers and soccer) had been shown by other authors (Candau et al., 1992; Ostojic and Ahmetovic, 2009). In contrast to these findings, many authors had shown post-seasonal iron deficiency leading to sports anaemia (Magazanik et al., 1988; Sinclair and Hinton, 2005).

Several authors note that not just blood iron concentration but also wider aspects of this microelement turnover should be analysed (Cazzola et al., 1985; Candau et al., 1992). Taking this into consideration, our study includes additional parameters that are typical of iron turnover in the body such as TIBC and UIBC.

TIBC is a useful indicator of iron metabolism in athletes. Decreased levels of this parameter have been observed in anaemia of chronic disease; on the other hand, increased levels of TIBC are typical for iron deficiency anaemia (Karagülle et al., 2013). In the tested field hockey players, a continuously but not statistically significant increase in TIBC values during the training programme was noted. Similar results were described by Magazanik et al. (1988) among females and males undergoing a 7 week endurance training programme. In contrast to our observations, in a study by Magazanik et al. (1988), the concentration of iron in the serum significantly decreased. Studies by Deruisseau et al. (2004) show that 3 weeks of strength training in untrained subjects did not have any direct influence on either iron serum concentration, transferrin or total iron binding capacity. The increase in TIBC concentration during physical training can therefore result from a previous decrease in iron concentration. The depletion of body iron content increases transferrin synthesis in the liver, which leads to an increase in the value of identified TIBC (Idzerda et al., 1986). Our study showed a simultaneous increase in TIBC and iron concentration. This may indicate that well-trained athletes exhibit a greater adaptation to long-term physical training. Their bodies responded to increased physical activity with transferrin synthesis, which supplemented the requirement for iron. Clearly, the concentration of iron alone is not sufficient to show the full changes in the turnover of this trace element of the entire human body. In addition, it can be stated that changes in iron metabolism depend on both the duration and type of physical activity and the fitness level of the training participants (Magazanik et al., 1988; Deruisseau et al., 2004).

Unsaturated iron binding capacity is used in clinical assessments as an indicator of the stage of iron deficiency anaemia or the consequence of some chronic diseases (Åsberg et al., 2013). In the literature, there is a lack of studies that use this indicator with reference to physical activity in humans. An animal study by Rowsey et al. (2009) showed a minor decrease in the value of this biochemical indicator among female rats who were subjected to physical activity in a running wheel over a few weeks of laboratory training. Our observations indicate that in well-trained athletes, blood proteins exhibit high levels of binding to iron during the training programme, which may be a result of increased transferrin synthesis in the liver. Similar changes after training have been shown by Magazanik et al. (1988). In our study, a simultaneous increase in iron concentration and UIBC was noted. This finding confirms our thesis that in well-trained athletes, only some early symptoms of anaemia without substantial changes in iron metabolism occur.

The lack of anaemia in our study does not exclude the need for monitoring of iron metabolism during physical activity. Considering a large number of biochemical indicators of anaemia can help prevent it. Even in well-trained athletes without systematic control, early symptoms of anaemia may progress to sports anaemia. In particular, because of regular menstrual iron losses, this phenomenon can affect female athletes.

## Conclusions

Iron metabolism in participants of various forms of physical activity, including competitive sports, is a very important and broad issue. A lack of iron metabolism control can lead to different types of anaemia, particularly sports anaemia. Monitoring of iron turnover should not be limited to this single biochemical indicator. As demonstrated in the present study, monitoring of additional variables such as UIBC, TIBC, haemoglobin, MCH and the erythrocyte count, is necessary to demonstrate global changes in iron metabolism in the human body. Furthermore, in addition to other studies, the present study showed that the direction of change in the metabolism of iron depends on both the duration and type of the physical activity and the fitness level of the training participants.

## Figures and Tables

**Table 1:** Mean values of somatic characteristics and training experience in the three phases of an annual training cycle.

Anthropometric data	Phase of an annual training cycle		

	Preparatory	Competitive	Transition
Body height [cm]±SD		177.1 ± 5.7	
Body mass [kg]±SD	73.5 ± 7.0	72.8 ± 7.6	73.7 ± 6.6
BMI [m^2^·kg^−1^]±SD	23.4 ± 1.5	23.2 ± 1.7	23.5 ± 1.4
Training experience [yrs]±SD		17.6 ± 4.4	

BMI – body mass index, SD – Standard deviation

**Table 2:** Mean values and differences between rest haematological variables associated with iron metabolism (haemoglobin concentration, erythrocyte count, MCH) in the three phases of an annual training cycle.

Haematological parameters	T1 [mean±SD]	T2 [mean±SD]	T3 [mean±SD]
Haemoglobin [mmol·L^−1^]	9.50 A ± 0.48	9.31^A^ ± 0.40	9.52 ± 0.45
Erythrocytes [10^12^·L^−1^]	4.87 ±0.27	4.92 ±0.26	4.99 ± 0.30
MCH [pg]	31.42^A^,^B^ ± 1.29	30.55^A^ ± 1.34	30.74^B^ ± 1.43

MCH – mean corpuscular haemoglobin, SD – Standard deviation, T1 – preparatory phase, T2 - competitive phase, T3 – transition phase

A Statistically significant difference between T1 and T2 (p<0.05)

B Statistically significant difference between T1 and T3 (p<0.05)
